# Developing a core competency framework for psychiatric pharmacists: a Europe-wide e-Delphi consensus study conducted by the European Society of Clinical Pharmacy Mental Health Special Interest Group

**DOI:** 10.1007/s11096-026-02213-0

**Published:** 2026-08-28

**Authors:** Ivana Tašková, Rosalind Gittins, Nikolaus Riesenhuber, Matej Stuhec, Martina Hahn, Nicole Šafářová, Daniela Fialová, Ita Fitzgerald

**Affiliations:** 1 Department of Clinical Pharmacy, Psychiatric Hospital Bohnice, Prague, Czech Republic; 2 Department of Social and Clinical Pharmacy, Faculty of Pharmacy, Charles University, Hradec Králové, Czech Republic; 3 School of Pharmacy, College of Health and Life Sciences, Aston University, Birmingham, UK; 4 College of Mental Health Pharmacy, Fareham, UK; 5 Department for Medicines Information and Clinical Pharmacy, Pharmacy of the University Hospital Vienna, Vienna, Austria; 6 Department of Pharmacology, Faculty of Medicine Maribor, University of Maribor, Maribor, Slovenia; 7 Department of Clinical Pharmacy, Ormoz Psychiatric Hospital, Ormoz, Slovenia; 8 Department of Psychiatry, Psychosomatics and Psychotherapy, University Medicine Frankfurt, Goethe University, Frankfurt am Main, Germany; 9 Department of Pharmacology and Clinical Pharmacy, Philipps-University Marburg, Marburg, Germany; 10 Department of Mental Health, varisano Hospital Frankfurt Hoechst, Frankfurt am Main, Germany; 11 National Institute of Mental Health, Klecany, Czech Republic; 12 Third Faculty of Medicine, Charles University, Prague, Czech Republic; 13 Department of Internal Medicine and Geriatrics, Charles University, Prague, Czech Republic; 14 Pharmacy Department, St Patrick’s Mental Health Services, Dublin, Ireland; 15 Pharmaceutical Care Research Group, School of Pharmacy, University College Cork, Cork, Ireland

**Keywords:** Clinical competence, Delphi technique, Hospital, Mental health services, Pharmacists, Pharmacy service, Psychiatric pharmacy

## Abstract

**Introduction:**

Consensus on essential clinical activities of psychiatric pharmacists is currently unavailable. Agreement on core non-clinical roles relating to research, education, leadership and management is also unavailable. Consensus on the core clinical and non-clinical functions of psychiatric pharmacists is urgently required to standardise the training of specialist pharmacists in psychiatric pharmacy and ensure the provision of high-quality patient care.

**Aim:**

The European Society of Clinical Pharmacy (ESCP) Mental Health Special Interest Group (SIG) aimed to develop a pan-European consensus-based core competency framework outlining clearly defined clinical and non-clinical competencies of psychiatric pharmacists.

**Method:**

A modified two-round e-Delphi method was used. Experts were purposively recruited and were eligible if they had at least three years of clinical experience in psychiatric pharmacy and were currently employed in a hospital-based clinical pharmacy service. Initial criteria were informed primarily by the International Pharmaceutical Federation Mental Health Care Handbook and elements of the College of Mental Health Pharmacy Advanced Pharmacist Mental Health Curriculum, and were adapted for the European context. Consensus was defined a priori as ≥ 80% agreement, a median score ≥ 3, and an interquartile range ≤ 1.

**Results:**

Of the 39 experts who agreed to participate, 32 (82.1%) completed Round 1 and 27 (69.2%) completed Round 2. Experts represented 13 European countries in Round 1 and 12 countries in Round 2. Thirty-three of the 45 initial criteria achieved consensus in Round 1. In Round 2, a further seven criteria achieved consensus, resulting in 40 final criteria for inclusion within the framework.

**Conclusion:**

The framework identifies 40 core criteria across seven domains of psychiatric pharmacy practice. This framework should be used to support the harmonised training of specialist psychiatric pharmacists, workforce development, and service planning across European healthcare systems. Future research should prioritise assessing factors affecting the effectiveness of implementation of criteria within each domain across clinical contexts and countries.

**Supplementary Information:**

The online version contains supplementary material available at https://doi.org/10.1007/s11096-026-02213-0.

## Impact statements

- This Europe-wide consensus framework provides a common reference for defining the clinical and non-clinical activities of psychiatric pharmacists.
- The framework may support curriculum development, workforce planning, and local review of psychiatric pharmacy services across European healthcare systems.
- Local implementation should account for differences in legislation, service organisation, and available resources, particularly for advanced and extended roles.

## Introduction

Clinical pharmacy services vary substantially across Europe in implementation, scope, and reimbursement [1, 2]. Competency development programmes are supported by European and international pharmacy guidance [3, 4], and competency frameworks define key professional behaviours, activities, and roles. Existing guidance, including the International Pharmaceutical Federation Mental Health Care Handbook and the European Statements of Hospital Pharmacy, does not explicitly define the core activities expected of psychiatric pharmacists [3, 4]. Consequently, no European consensus-based framework currently describes these activities across mental healthcare settings, limiting pharmacist development, service optimisation, and potential improvements in patient outcomes [5].

Previous studies of European clinical pharmacy standards have identified the absence of a national competency framework informing postgraduate education and training as contributing to deficits in pharmacist education, including essential clinical skills and those required for advanced specialist practice [5]. Although many European countries have developed clinical pharmacy specialisations [2], the lack of comparable frameworks results in inconsistent roles and recognition of clinical pharmacists within healthcare systems [1, 2]. Even where general practice frameworks are available, implementation and reimbursement challenges may limit the integration of clinical pharmacy services [1, 2].

Psychiatric pharmacy is a specialist area of clinical pharmacy focussed on the safe and effective use of medicines in mental illnesses [1]. Across Europe, pharmacists’ roles are integrated within multidisciplinary teams, although implementation varies, particularly between inpatient and outpatient settings [1, 5, 6]. Prior research has identified heterogeneity in clinical practice across Europe [1, 2]. Several countries have developed national models for clinical pharmacy practice outlining pharmaceutical care processes, service implementation and integration into healthcare systems [7–11]. However, they are not specific to psychiatric pharmacy and do not define core clinical and non-clinical (i.e., research, education, leadership and governance) activities applicable across European settings [7–12]. Whilst the UK has developed curriculum frameworks describing capabilities, domains, and learning outcomes [12], European consensus on essential activities of psychiatric pharmacists is required to standardise specialist training and ensure high-quality, consistent patient care.

### Aim

The European Society of Clinical Pharmacy (ESCP) Mental Health Special Interest Group (SIG) aimed to develop a pan-European consensus-based core competency framework outlining clearly defined clinical and non-clinical competencies of psychiatric pharmacists.

## Method

### Study design

This study used a modified electronic Delphi (e-Delphi) consensus method [13–16]. It was considered modified because the initial criteria were developed by the research team before Round 1 from existing professional resources and expert input, rather than generated by panellists. The Delphi method is widely used in health services research to develop consensus-based frameworks and professional standards and systematically refines expert judgements through multiple questionnaire rounds [13–16]. Each round was informed by the previous round through summary statistics and aggregate comments until consensus was achieved [14, 17, 18]. The study involved two online questionnaires conducted using Google Forms. The reporting of the competency framework and Delphi process was reviewed against the CONFERD-HP and DELPHISTAR guidelines, respectively.

### Development of the initial criterion list and questionnaire

Competencies were operationalised as observable activities and roles relevant to European psychiatric pharmacy practice. The initial criteria were informed primarily by the FIP Mental Health Care Handbook [3], supplemented by relevant elements from the CMHP Advanced Pharmacist Mental Health Curriculum [12] and published literature [1, 19–21]. Related criteria were grouped into seven domains and refined by the research team, including four members of the ESCP Mental Health SIG. One member of the ESCP Deprescribing SIG independently reviewed the criteria. Finally, credentialed members of the CMHP provided external expert input.

**Fig. 1:**
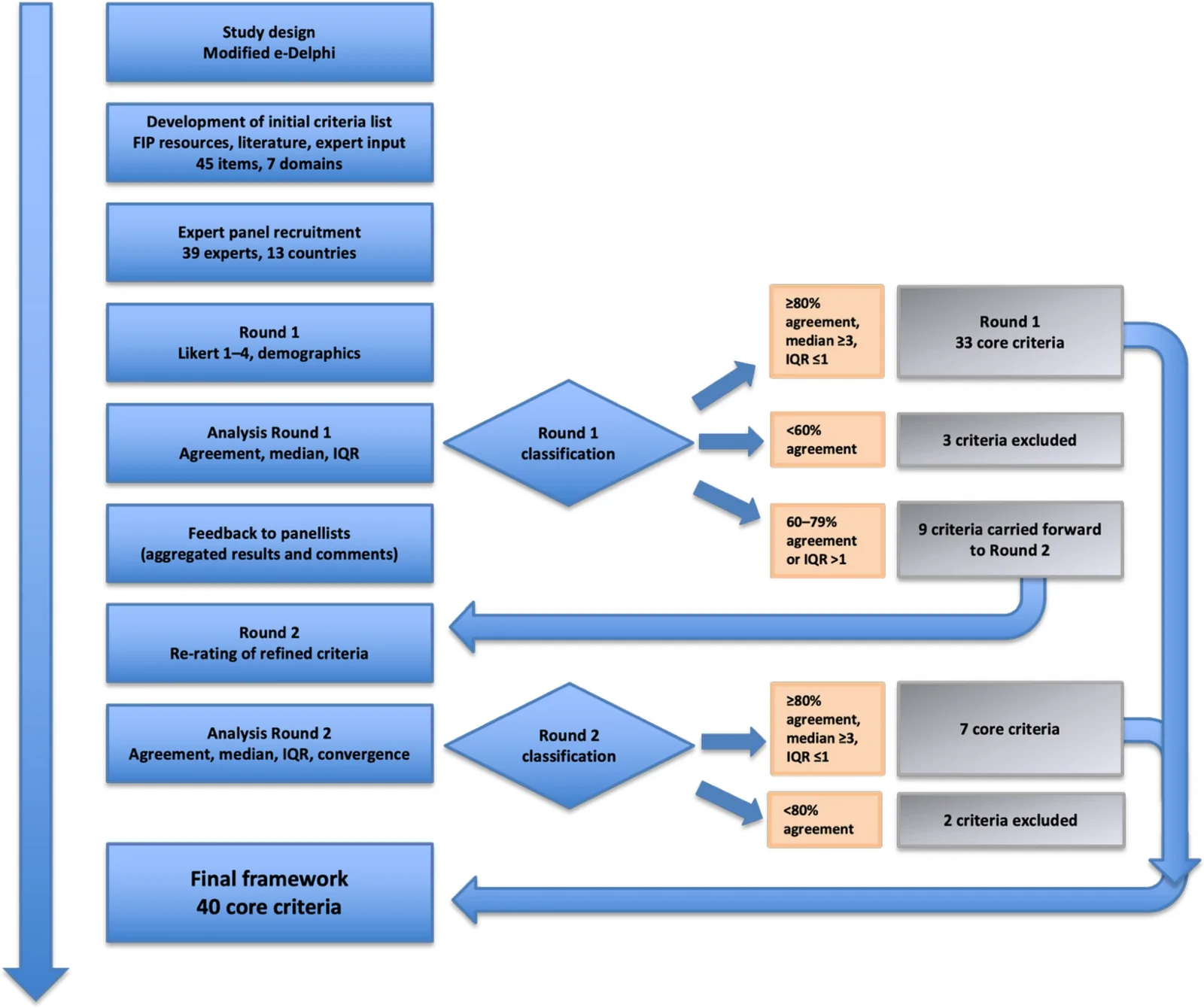
Flowchart of the modified e-Delphi process used in developing a consensus-based framework of core activities of psychiatric pharmacists across Europe

In total, 45 competencies and activities were developed across seven domains: (1) Medicines optimisation, (2) Physical health monitoring and interventions, (3) Person-centred care and counselling, (4) Interprofessional collaboration and care coordination, (5) Advanced and extended roles, (6) Administrative and governance activities, and (7) Research, education, and service development. The full list of initial criteria is contained within the Supplementary Material S1. The complete questionnaire distributed in Round 1 is available in Supplementary Material S2. An overview of the modified e-Delphi process, including criteria development, panel recruitment, consensus procedures, and criterion progression across rounds, is presented in Fig. 1.

### Expert panel recruitment

Experts were defined a priori by the ESCP Mental Health SIG through consensus discussions as clinical pharmacists with demonstrated experience in clinical pharmacy practice in mental health. For the purposes of this study, an expert was defined as a pharmacist who had: (1) a minimum of three years of clinical experience in psychiatric pharmacy practice and (2) current employment in a hospital-based clinical pharmacy service. Given the specialised nature of psychiatric clinical pharmacy, a purposive expert sampling strategy was used. A minimum target of 15 experts from at least seven European countries was agreed pragmatically to ensure diversity of professional and geographical perspectives while maintaining a manageable Delphi panel. This approach is consistent with Delphi methodology, where expert panels are typically purposively selected and may remain relatively small in specialised fields [22, 23]. Both rounds of the e-Delphi consensus voting were distributed between February and March 2026. Each round remained open for three weeks. Only responses submitted within the prespecified periods were included in the analyses; two responses received after the closure of Round 2 were excluded. No financial or non-financial compensation was provided for participation.

### Conducting the e-Delphi—rounds 1 and 2

In Round 1, participants completed a demographic questionnaire describing their professional background and experience across psychiatric pharmacy, academia and education. Only the principal investigator (IT) had access to the participant details and their individual responses. Other members of the research team reviewed aggregate responses only. Each expert was assigned a unique identification code, known only to the participant and the principal investigator, allowing responses to be linked across Delphi rounds while preserving confidentiality. Participants could opt to be named in the Acknowledgements; only those who provided explicit consent are listed. Participants were subsequently asked to evaluate the preliminary 45 clinical and non-clinical activities and competencies and rate their agreement with the essential nature of each criterion using a four-point Likert scale (1—strongly disagree to 4—strongly agree).

A summary of criterion-level revisions is provided in Supplementary Material S3. In the second round, participants received aggregated results from Round 1 for each criterion (percentage agreement, median score, and IQR). Participants were asked to vote again regarding their level of agreement with each statement. Participants could identify their previous responses and compare them with the overall panel results. The feedback also indicated which criteria had reached consensus and which had been excluded.

### Data analysis

Data were analysed descriptively after each Delphi round. For each criterion, the percentage agreement (ratings of 3 or 4), median score, and interquartile range (IQR) were calculated. The two-round design and all decision rules were specified before data collection in the study documentation submitted for ethics approval. Consensus required ≥ 80% agreement, a median score ≥ 3, and an IQR ≤ 1. The 80% threshold was selected to require strong agreement; items with 60–79% agreement or IQR > 1 were revised for Round 2, whereas < 60% agreement indicated insufficient support [14–18]. At the end of each domain, an optional free-text field allowed participants to suggest missing activities or revisions. Comments were reviewed descriptively and informed revisions for Round 2; no new items were added, and no formal qualitative analysis, subgroup analysis, or response weighting was undertaken. Revisions focused on improving clarity, specifying scope of practice, and ensuring applicability across diverse European healthcare systems, while preserving the original conceptual intent of each activity. Wording was adjusted to reflect multidisciplinary collaboration, variability in national legislation, and differences in service organisation.

Stability of responses between rounds was assessed by comparing the median score and IQR for each criterion. Responses were considered stable when the median remained unchanged or varied by no more than one point, and the IQR did not increase. Convergence between rounds was investigated by analysing shifts towards greater consensus and decreased variability. After Round 2, items meeting all predefined criteria were classified as core regardless of domain; all other items were excluded.

### Ethics approval

Prior to study commencement, ethical approval for the study was obtained from the Research and Ethics Committee of the Faculty of Pharmacy, Charles University, Czech Republic (Reference No. UKFaF/707308/2025, approved on 3 December 2025). Electronic informed consent was obtained at the start of Round 1; participants could not proceed without confirming consent.

## Results

Thirty-nine experts from 13 European countries agreed to participate. Thirty-two (82.1%) completed Round 1, representing 13 countries. Participation in Round 2 included 27 (69.2%) participants from 12 countries. Experts represented a broad spectrum of European countries and healthcare systems and had varying levels of clinical expertise. An overview of demographics of respondents is provided in Table 1.

**Table 1:** Overview of respondent characteristics (n = 32)

Characteristic	n (%) or value
Countries represented	13
Age, median (range), years	45.5 (30–73)
*Professional experience in pharmacy*	
4–10 years	6 (18.8)
11–14 years	5 (15.6)
≥ 15 years	21 (65.6)
*Experience in clinical pharmacy in psychiatry*	
3–10 years	15 (46.9)
11–14 years	9 (28.1)
≥ 15 years	8 (25.0)
*Primary workplace*	
Hospital (inpatient)	20 (62.5)
Hospital and ambulatory	12 (37.5)
*Actively involved in research*	
Yes	18 (56.3)
*Peer‑reviewed publications in last 5 years*	
0	8 (25.0)
1–3	12 (37.5)
4–10	8 (25.0)
> 10	4 (12.5)
*Participation in committees/working groups*	
National committee/working group	12 (37.5)
International committee/working group	7 (21.9)
Steering committee/board member	4 (12.5)
None	13 (40.6)
*Membership in professional societies*	
National pharmacy society	23 (71.9)
National psychiatry/mental health society	7 (21.9)
International pharmacy society	5 (15.6)
International psychiatry/mental health society	3 (9.4)
*Role in education and training*	
Undergraduate teaching	18 (56.3)
Postgraduate/specialization training	20 (62.5)
Continuing professional development (CPD)	19 (59.4)
Supervision/mentoring	14 (43.8)
*Typical involvement in education and training activities (hours/year)*	
Occasional (< 10 h/year)	7 (21.9)
Regular (10–49 h/year)	19 (59.4)
Extensive (≥ 50 h/year)	6 (18.8)

Participants could select more than one response for committee or working-group participation, professional society membership, and education and training roles

**Fig. 2:**
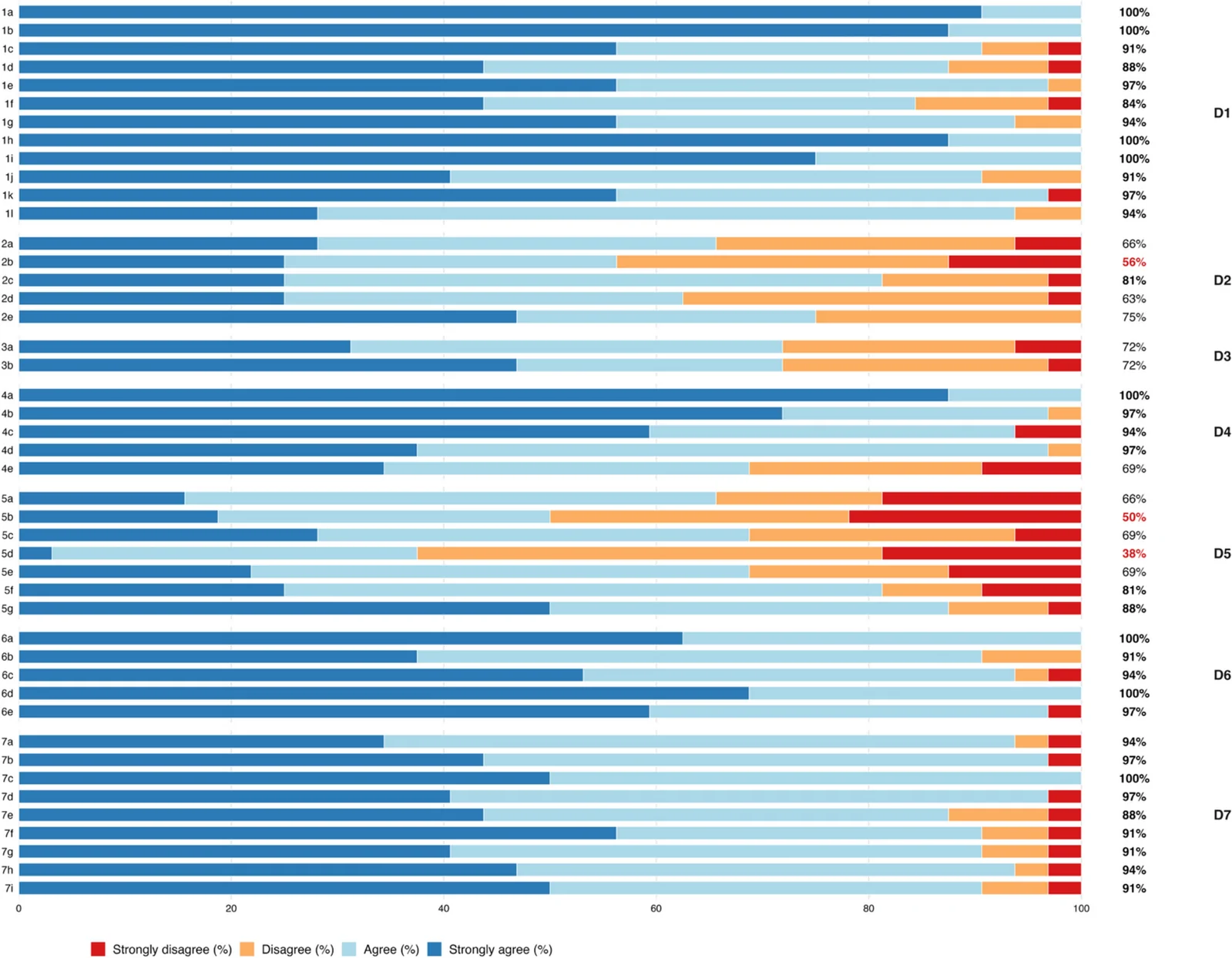
Likert-scale response distributions by criterion in Round 1. Item codes correspond to the Round 1 questionnaire provided in Supplementary Material S2. Stacked bars represent the distribution of responses across the four Likert categories. Percentages indicate agreement (Agree + Strongly agree). Bold values indicate criteria that met the predefined consensus criteria. Non-bold black values indicate criteria carried forward to Round 2 due to moderate agreement or high variability. Red values indicate criteria excluded after Round 1. Domain codes are: D1, Medicines optimisation; D2, Physical health monitoring and interventions; D3, Person-centred care and counselling; D4, Interprofessional collaboration and care coordination; D5, Advanced and extended roles; D6, Administrative and governance activities; and D7, Research, education, and service development

In Round 1, 33 of 45 criteria (73.3%) achieved consensus; 9 criteria (20.0%) associated with moderate agreement (60–79%) or high variability were carried forward to Round 2. Three criteria (6.7%) were excluded. Eight criteria achieved 100% agreement in Round 1; median scores ranged from 3.5 to 4 and IQRs from 0 to 1. Of the 9 criteria carried forward to Round 2, 7 (77.8%) reached consensus following refinement, while 2 criteria remained below the predefined threshold. Thus, 40 out of 45 criteria reached final consensus following two rounds of voting. Response distributions for individual criteria in Round 1 are presented in Fig. 2.

**Fig. 3:**
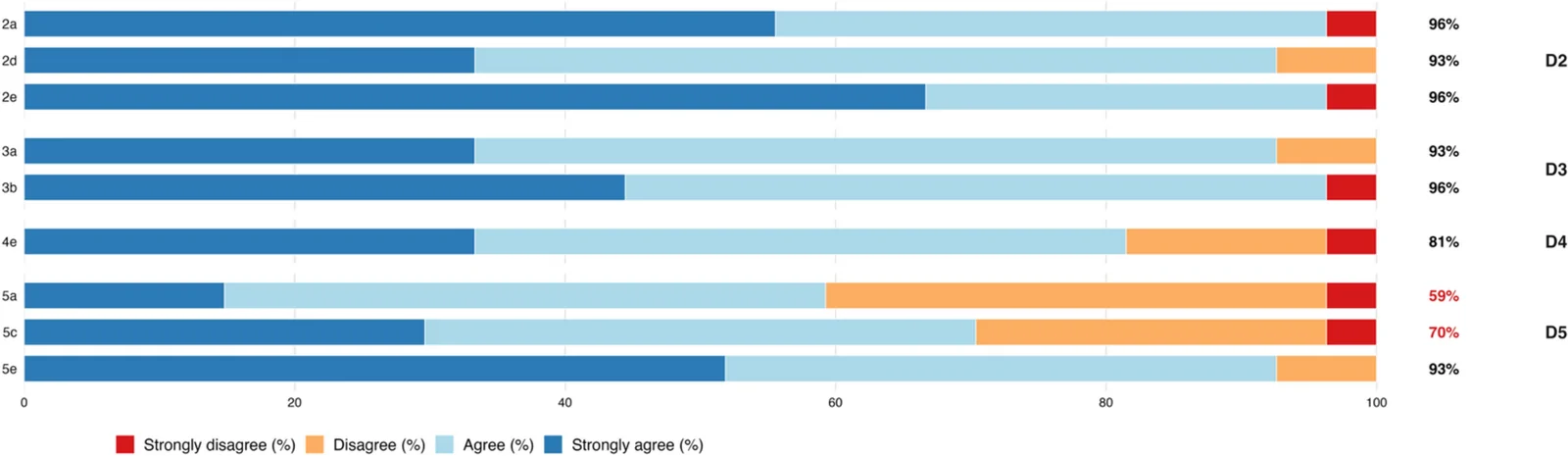
Likert-scale response distributions for criteria re-evaluated in Round 2. Item codes and the corresponding revised criteria are provided in Supplementary Material S3. Stacked bars represent the distribution of responses across the four Likert categories. Percentages indicate agreement (Agree + Strongly agree). Bold values indicate criteria that met the predefined consensus criteria; red values indicate criteria that did not meet the predefined consensus threshold. Domain codes shown are: D2, Physical health monitoring and interventions; D3, Person-centred care and counselling; D4, Interprofessional collaboration and care coordination; and D5, Advanced and extended roles

Response distributions for criteria re-evaluated after refinement in Round 2 are presented in Fig. 3.

Detailed item-level results, including agreement percentages, median scores and IQRs for Rounds 1 and 2, are provided in Supplementary Material S4. Among the nine criteria re-evaluated in Round 2, median scores remained stable or changed by no more than one point, while IQRs generally decreased or remained unchanged. The final set of consensus-based criteria that met the predefined consensus criteria is presented in Table 2.

**Table 2:** Final consensus-based core roles and activities of psychiatric pharmacists by domain and original Delphi item code

No.	Final criterion	Item code
*Domain 1: Medicines Optimisation*		
1.	Conduct comprehensive medication reviews for individuals with mental illness including pharmacotherapy across specialties. This includes reviewing the appropriateness of the following prescribing parameters relating to each medication: (i) indication, including optimised selection within the drug class; (ii) duration of treatment; (iii) potential for drug-drug and drug-disease interactions; (iv) proactive management, including prevention of adverse drug reactions and other medicine-related problems	1a
2.	Prioritise structured medication review in older adults and others with multimorbidities (e.g., cardiac, renal, or hepatic impairment), polypharmacy, frailty, or use of higher-risk psychotropic medicines (e.g., high-dose antipsychotics, lithium, anticholinergic drugs, benzodiazepines) to reduce treatment burden, prevent medication-related harm, and improve quality of life	1b
3.	Use validated tools (e.g., Beers, STOPP/START) within comprehensive medication reviews to recommend safer alternatives and minimise the risk of prescribing cascades	1c
4.	Provide medication reviews within both primary and secondary care settings	1d
5.	Ensure medication reconciliation is completed. Medicines reconciliation includes ensuring an accurate and complete medication history is taken, identifying and resolving discrepancies, and reviewing pharmacotherapy in collaboration with the multidisciplinary team. Reconciliation may be repeated in community, primary care settings, and at transition points with follow-up reviews added as needed to ensure safe and coordinated care delivery	1e
6.	Provide seamless care, including during admission and transitions of care, including discharge	1f
7.	Support adherence through tailored interventions and facilitate shared decision-making within consultations (e.g., support with long-acting injectable antipsychotic administration, management of adverse effects, etc.)	1g
8.	Manage clinically significant drug interactions (including drug-drug, drug-gene, and drug-disease, etc.)	1h
9.	Optimise use of high-risk psychotropics (including clozapine and long-acting injectable antipsychotics/high-dose antipsychotics), including adherence to physical health monitoring requirements	1i
10.	Optimise the prescribing of psychotropic medication using pharmacokinetic and pharmacogenomic tools, as appropriate	1j
11.	Make therapeutic substitutions where appropriate (e.g., formulary limitations, medicine shortages) ensuring equivalent efficacy and safety	1k
12.	Provide interventions at multiple points of care, including admission and transitions of care, including discharge	1l
*Domain 2: Physical Health Monitoring and Interventions*		
13.	Recognise and flag physical–mental health interdependencies relevant to pharmacotherapy (e.g., medication-related metabolic effects) and recommend appropriate assessment, review, or referral within the multidisciplinary team	2a
14.	Recommend and implement physical health and lifestyle interventions for people with mental illness, including smoking cessation services, weight management, preventive screening programmes, and protocols supporting individuals with somatic comorbidities or medication-related risks (e.g., clozapine)	2c
15.	Use relevant tools, where available, to support identification of physical health risks and to guide recommendations for modifiable lifestyle factors	2d
16.	Support monitoring and optimisation of chronic conditions in people receiving psychotropic medication by identifying medication-related risks, prompting appropriate follow-up, and collaborating with the multidisciplinary team	2e
*Domain 3: Person-Centred Care and Counselling*		
17.	Provide person-centred pharmaceutical care in relation to medicines, involving individuals (and, where appropriate, families or carers) in shared decision-making about pharmacotherapy within the multidisciplinary team	3a
18.	Provide medication counselling and medicines-related education to the people using them, including families or carers, supporting understanding, adherence, and shared treatment goals, while respecting individual cognitive and health literacy needs	3b
Domain 4: Interprofessional Collaboration and Care Coordination		
19.	Integrate into multidisciplinary teams to provide expert pharmaceutical input	4a
20.	Collaborate across care settings with pharmacists (hospital, community, general practice) and other healthcare professionals (e.g., psychiatrists, GPs, nurses, geriatricians, social workers, occupational therapists) to ensure safe and effective treatment and continuity of care, particularly during transitions of care (including admission, discharge, and primary care)	4b
21.	Document and communicate care plans, actions, and outcomes in a timely manner to support collaborative decision-making among the team	4c
22.	Engage in professional networks and representative groups at local, national, and international levels to support service development and professional advocacy	4d
23.	Contribute, where relevant and feasible, to discussions on funding or reimbursement models to demonstrate the value of mental health pharmacy services within the healthcare system	4e
*Domain 5: Advanced and Extended Roles*		
24.	Facilitate medicines-focused group-based interventions (e.g., education about psychotropic medicines, adherence support, and management of adverse effects), where appropriate within scope of practice	5e
25.	Offer community post-discharge services, including medicines reconciliation, outpatient medication reviews, and adherence support	5f
26.	Optimise the use of new technologies to support medication-related decision-making (e.g., clinical decision-support systems, electronic prescribing tools, medication safety software, pharmacogenomic decision tools)	5g
Domain 6: Administrative and Governance Activities		
27.	Manage referrals and monitor high-risk medications to ensure safe use	6a
28.	Conduct audits of prescribing practices and adherence to best practice standards	6b
29.	Contribute to formulary decision-making regarding approved medications	6c
30.	Develop and review clinical practice guidelines, clinical pharmacy guidance, and decision-making tools to support safe and effective prescribing in mental health	6d
31.	Implement local and national practice recommendations, where relevant (e.g., medication-related monitoring protocols) across clinical teams and within the service	6e
*Domain 7: Research, Education, and Service Development*		
32.	Lead or participate in research, including clinical trials relevant to pharmacy services in mental health	7a
33.	Collaborate within academic and professional networks to conduct research relevant to mental health pharmacy	7b
34.	Provide education and mentorship to advance evidence-based mental health pharmacy practice	7c
35.	Undertake quality improvement and service development projects	7d
36.	Establish and monitor quality indicators to demonstrate the effectiveness of mental health pharmacy services	7e
37.	Teach clinical pharmacy in mental health, including within undergraduate and postgraduate programmes, and among pharmacists and other healthcare professions	7f
38.	Supervise undergraduate research, including thesis/dissertation supervision	7g
39.	Conduct projects in mental health pharmacy to support innovation, service development, and reimbursement models	7h
40.	Disseminate results associated with service evaluation to relevant stakeholders, including healthcare leaders, policymakers, and the public	7i

Across the 40 criteria included in the final framework, item-level agreement ranged from 81.25% to 100%. All included criteria met the predefined consensus thresholds of ≥ 80% agreement, a median score ≥ 3, and an IQR ≤ 1. Domain-level summaries of the final included criteria are presented in Table 3.

**Table 3:** Domain-level summary of the final included criteria

Domain	Final core criteria number	Item-level agreement (%), range	Median score, range	IQR, range
D1: Medicines optimisation	12	84.38–100.00	3–4	0–1
D2: Physical health monitoring and interventions	4	81.25–96.30	3–4	1
D3: Person-centred care and counselling	2	92.59–96.30	3	1
D4: Interprofessional collaboration and care coordination	5	81.48–100.00	3–4	0–1
D5: Advanced and extended roles	3	81.25–92.59	3–4	1
D6: Administrative and governance activities	5	90.63–100.00	3–4	1
D7: Research, education, and service development	9	87.50–100.00	3–4	1
Total	40	81.25–100.00	3–4	0–1

Agreement, median, and IQR values represent item-level ranges within each domain. Round 1 results were used for criteria finalised after Round 1, and Round 2 results were used for criteria re-evaluated in Round 2. Excluded criteria were not included

## Discussion

This study outlines the development process of the first European core competency framework outlining core clinical and non-clinical competencies for specialist psychiatric pharmacists working across clinical care settings. Forty criteria were included in the final framework across seven domains of practice spanning clinical, leadership, research, education and governance activities.

Similar to established international and national frameworks, this framework covers both clinical and non-clinical practice [3, 4, 8, 12]. It differs by defining consensus-based, psychiatry-specific activities applicable across European settings. Agreement was high across most domains but more variable for advanced roles, particularly prescribing.

Consensus across experts was high for medicines optimisation activities, including medication review, medicines reconciliation, interaction management, and optimisation of high-risk psychotropics. As expected, consensus regarding their inclusion as core competencies reflects the central role of identifying medication-related problems and optimising pharmacotherapy in psychiatric pharmacy practice, particularly given the frequent complexity of psychotropic treatment and occurrence of multimorbidity among people living with mental illnesses [20, 21]. Our findings suggest that these routine clinical activities are conceptualised among pharmacists as core and foundational activities from which other areas of practice can develop.

High agreement was also observed for person-centred care and interprofessional collaboration, consistent with international and European pharmacy guidance [3, 4]. Both person-centred care criteria reached consensus, supporting shared decision-making and medicines-related education as core elements of psychiatric pharmacy practice. All five interprofessional collaboration criteria were also included, covering integration within multidisciplinary teams, collaboration across care settings, communication of care plans, professional networking, and engagement with funding or reimbursement discussions. Within advanced and extended roles, the panel supported group-based medicines interventions, post-discharge services, and digital decision-support technologies, which are also described in recent literature on outpatient mental health pharmacy services [6]. Prescribing-related activities remained more contested, as discussed below. All five governance criteria reached consensus, including management of referrals and high-risk medicines, audit, formulary input, guideline development, and implementation of practice recommendations.

Despite frequent discussion regarding the expansion of pharmacist roles to include prescribing practices—both independent and dependent—prescribing-related criteria did not meet the predefined consensus thresholds whereby agreement regarding these activities being core roles among psychiatric pharmacists was variable. Independent prescribing was excluded after Round 1, while dependent prescribing remained below the agreement threshold after Round 2. Variability regarding prescribing as a core activity of pharmacists may reflect differences in national legislation, training pathways, scope of practice and service organisation rather than a judgement that pharmacist prescribing is unimportant. Variation in funding models, capacity and ability to provide core clinical services may also explain the de-prioritisation of prescribing roles seen among experts here. Exploring factors underlying the variability of agreement regarding its core nature will be essential for prioritisation within future research, particularly if the widely discussed role of independent prescribing is to become routinely established.

Recent evidence from the Netherlands indicates that pharmacist prescribing is not yet routinely established [24]. This is despite the benefits demonstrated in clinical settings. For example, in Slovenia, a collaborative pharmacist prescribing model in primary care has been associated with improvements in patient-reported and clinical outcomes, including outcomes related to depression [25]. Prescribing roles may become more widely applicable as national legislation and training pathways evolve; however, our findings suggest that substantial preparatory work to understand the current barriers to integration of both dependent and independent pharmacist prescribing is required before such practices are effectively integrated into core psychiatric pharmacy practice across Europe.

Although all four criteria included within the physical health monitoring and associated interventions domain were agreed across experts as core competencies, item-level agreement varied within this domain. The four criteria addressed recognition of physical–mental health interdependencies, recommendation and implementation of physical health and lifestyle interventions, use of relevant tools to identify physical health risks, and collaborative monitoring and optimisation of chronic conditions. Item-level agreement was lowest for recommending and implementing physical health and lifestyle interventions (81.25%), whereas the other three criteria achieved 92.59–96.30% agreement. Observed variability across domains may reflect differences in how physical health monitoring and management services are integrated into psychiatric pharmacists’ scopes of practice across European healthcare systems. The inclusion of physical health monitoring and intervention as core competencies is particularly prudent within psychiatry given the mortality gap of 10–20 years experienced by people with mental illness being predominantly explained by premature mortality related to cardiometabolic disease and the contribution of often essential psychotropic medicines to adverse cardiometabolic outcomes, with risk exposure occurring typically early in life [26].

All nine criteria within the research, education, and service development domain met the predefined consensus thresholds and were thus classified as core competencies. This finding is consistent with professional frameworks describing clinical pharmacy as both a field of professional practice and research and which identify education, research, and professional development as essential components of advanced pharmacy practice [4, 27, 28]. Recent studies continue to identify research skills, engagement in research, and further education as development needs among pharmacists practising at advanced or specialist levels [29–31]. This gap between professional frameworks and real-world practice is not explained by a lack of prioritisation among pharmacists, as demonstrated by our findings. Rather, prior research has identified the absence of sufficient time, dedicated funding streams and advanced levels of training in skillsets relevant to conducting robust research as key barriers to engagement in research and education. Further research should therefore assess the implementation of these criteria and identify barriers and facilitators across healthcare systems.

Although developed primarily by hospital-based experts, the framework may inform psychiatric clinical pharmacy services across European care settings, subject to local adaptation.

### Limitations

The results of this study and the consensus framework should be considered in the context of several potential limitations. Firstly, recruitment through professional networks, the predominance of hospital-based pharmacists, and attrition from 32 to 27 participants may limit representativeness. Attrition may also have affected criteria with agreement close to the consensus threshold, although the direction and extent of this effect cannot be determined. This may limit the transferability of findings to community, primary care, outpatient, or non-hospital psychiatric pharmacy settings. Secondly, the initial criteria list was informed by the FIP Mental Health Care Handbook and subsequently adapted for the specialty of psychiatry, rather than based on a systematic assessment and critical appraisal of all available pharmacy practice frameworks internationally. Although we put several mechanisms in place to ensure the criteria list was robust and subject-specific, the results of a systematic appraisal of all available frameworks may have informed the final list of criteria circulated to experts for consensus voting. This was not within the capacity of the ESCP Mental Health SIG at the time of project development. In future work we plan to conduct such assessments and publish revised versions of the competency framework, as needed. Thirdly, the consensus framework was developed based on the experience and expertise of European psychiatric pharmacists. Thus, it should not be assumed that the framework can be extrapolated to psychiatric pharmacy practice in non-European settings. Adaptation of this framework for use within other settings should be the focus of future research by similar specialist interest groups outside of Europe. Some criteria overlap in scope because they were evaluated separately; future revisions should assess whether closely related items can be consolidated or more clearly differentiated. Finally, this report focuses on consensus regarding the essential nature of the criteria. Implementation, feasibility, and prioritisation address a distinct research question and are being analysed separately for a subsequent manuscript. Inclusion of a criterion should not be interpreted as evidence that the activity is uniformly implemented across European healthcare systems.

## Conclusion

This study provides the first European consensus-based core competency framework for specialist psychiatric pharmacists working across clinical care settings. Forty criteria across seven domains of practice were agreed through expert consensus. The findings demonstrate broad agreement that psychiatric pharmacy practice is centred on medicines optimisation and collaborative clinical care. Criteria relating to dependent and independent pharmacist prescribing did not meet the predefined consensus thresholds, whereas all research, education, and service development criteria were classified as core.

## Supplementary Information

Below is the link to the electronic supplementary material.

Supplementary file1 (PDF 224 KB)

Supplementary file2 (PDF 139 KB)

Supplementary file3 (PDF 94 KB)

Supplementary file4 (PDF 229 KB)

## Data Availability

The data supporting the findings of this study are provided in the supplementary materials. Additional anonymised data are available from the corresponding author on reasonable request.
