# Supplementary material for: Developing a core competency framework for psychiatric pharmacists: a Europe-wide e-Delphi consensus study conducted by the European Society of Clinical Pharmacy Mental Health Special Interest Group
Source: Int J Clin Pharm. 2026 Aug 28;48(6):2360–72. doi: 10.1007/s11096-026-02213-0 (PMC13731694; doi:10.1007/s11096-026-02213-0)
Supplement: Supplementary file 1 [file 11096_2026_2213_MOESM1_ESM.pdf]

Supplementary Material S1. Initial set of proposed items included in the first round of the Delphi survey.		
**Domain**	**Number**	**Criteria**
**1. Medicines Optimisation**	**1.**	a. Conduct comprehensive medication reviews for individuals with mental illness including pharmacotherapy across specialties. This includes reviewing the appropriateness of the following prescribing parameters relating to each medication: (i) indication, including optimised selection within the drug class; (ii) duration of treatment; (iii) potential for drug-drug and drug-disease interactions; (iv) proactive management, including prevention of adverse drug reactions and other medicine-related problems.
	**2.**	b. Prioritise structured medication review in older adults and others with multimorbidities (e.g., cardiac, renal, or hepatic impairment), polypharmacy, frailty, or use of higher-risk psychotropic medicines (e.g., high-dose antipsychotics, lithium, anticholinergic drugs, benzodiazepines) to reduce treatment burden, prevent medication-related harm, and improve quality of life.
	**3.**	c. Use validated tools (e.g., Beers, STOPP/START) within comprehensive medication reviews to recommend safer alternatives and minimise the risk of prescribing cascades.
	**4.**	d. Provide medication reviews within both primary and secondary care settings.
	**5.**	e. Ensure medication reconciliation is completed. Medicines reconciliation includes ensuring an accurate and complete medication history is taken, identifying and resolving discrepancies, and reviewing pharmacotherapy in collaboration with the multidisciplinary team. Reconciliation may be repeated in community, primary care settings, and at transition points with follow-up reviews added as needed to ensure safe and coordinated care delivery.
	**6.**	f. Provide seamless care, including during admission and transitions of care, including discharge.
	**7.**	g. Support adherence through tailored interventions and facilitate shared decision-making within consultations (e.g., support with long-acting injectable antipsychotic administration, management of adverse effects, etc.).
	**8.**	h. Manage clinically significant drug interactions (including drug-drug, drug-gene, and drug-disease, etc.)
	**9.**	i. Optimise use of high-risk psychotropics (including clozapine and long-acting injectable antipsychotics/high-dose antipsychotics), including adherence to physical health monitoring requirements.
	**10.**	j. Optimise the prescribing of psychotropic medication using pharmacokinetic and pharmacogenomic tools, as appropriate.
	**11.**	k. Make therapeutic substitutions where appropriate (e.g., formulary limitations, medicine shortages) ensuring equivalent efficacy and safety.
	**12.**	l. Provide interventions at multiple points of care, including admission and transitions of care, including discharge.
**2. Physical Health Monitoring and Interventions**	**13.**	a. Recognise and address physical–mental health interdependencies (e.g., depression linked to hormonal imbalance) and recommend appropriate interventions (e.g., tests or reviews).
	**14.**	b. Conduct physical health checks for people on psychotropic medication, including weight, waist circumference, blood pressure, pulse, and necessary blood tests at recommended intervals, and act on any out-of-range results (e.g., provide appropriate management, recommend referral to an alternative clinician).
	**15.**	c. Recommend and implement physical health and lifestyle interventions for people with mental illness, including smoking cessation services, weight management, preventive screening programmes, and protocols supporting individuals with somatic comorbidities or medication-related risks (e.g., clozapine).
	**16.**	d. Use relevant tools to support recommendations for physical health interventions (e.g., the Lester Tool) and modifiable lifestyle risk factors (such as smoking and substance use).
	**17.**	e. Monitor chronic diseases in at-risk individuals to minimise the impact of psychotropic medication on disease burden and to support optimisation of chronic conditions (e.g., diabetes, hypertension or obesity).
**3. Person-Centred Care and Counselling**	**18.**	a. Provide personalised and person-centred pharmaceutical care, including involving people and their families or carers as active partners in planning, monitoring, and decision-making throughout treatment.
	**19.**	b. Offer clear medication counselling and psychoeducation to people and their families or carers, supporting understanding, adherence, and shared treatment goals while accommodating individual cognitive or health literacy needs.
**4. Interprofessional Collaboration and Care Coordination**	**20.**	a. Integrate into multidisciplinary teams to provide expert pharmaceutical input
	**21.**	b. Collaborate across care settings with pharmacists (hospital, community, general practice) and other healthcare professionals (e.g., psychiatrists, GPs, nurses, geriatricians, social workers, occupational therapists) to ensure safe and effective treatment and continuity of care, particularly during transitions of care (including admission, discharge, and primary care).
	**22.**	c. Document and communicate care plans, actions, and outcomes in a timely manner to support collaborative decision-making among the team.
	**23.**	d. Engage in professional networks and representative groups at local, national, and international levels to support service development and professional advocacy.
	**24.**	e. Contribute to discussions on reimbursement models by working with commissioners, insurers and within healthcare systems to demonstrate the value of pharmacy services.
**5. Advanced and Extended Roles**	**25.**	a. Undertake dependent (collaborative) prescribing under shared care agreements with physicians.
	**26.**	b. Undertake independent prescribing within a defined scope of practice.
	**27.**	c. Develop pharmacist-led services for people with mental illnesses, including support for more severe and enduring mental illnesses (e.g., clozapine clinics).
	**28.**	d. Provide direct behavioural or mental health support in exceptional circumstances (e.g., crisis situations), where additional specialist training is available.
	**29.**	e. Facilitate group-based interventions (e.g., therapeutic groups for people with mental illnesses) to encourage discussion and identify medication-related problems.
	**30.**	f. Offer community post-discharge services, including medicines reconciliation, outpatient medication reviews, and adherence support.
	**31.**	g. Optimise the use of new technologies to support medication-related decision-making (e.g., clinical decision-support systems, electronic prescribing tools, medication safety software, pharmacogenomic decision tools).
**6. Administrative and Governance Activities**	**32.**	a. Manage referrals and monitor high-risk medications to ensure safe use.
	**33.**	b. Conduct audits of prescribing practices and adherence to best practice standards.
	**34.**	c. Contribute to formulary decision-making regarding approved medications.
	**35.**	d. Develop and review clinical practice guidelines, clinical pharmacy guidance, and decision-making tools to support safe and effective prescribing in mental health.
	**36.**	e. Implement local and national practice recommendations, where relevant (e.g., medication-related monitoring protocols) across clinical teams and within the service.
**7. Research, Education and Service Development**	**37.**	a. Lead or participate in research, including clinical trials relevant to pharmacy services in mental health.
	**38.**	b. Collaborate within academic and professional networks to conduct research relevant to mental health pharmacy.
	**39.**	c. Provide education and mentorship to advance evidence-based mental health pharmacy practice.
	**40.**	d. Undertake quality improvement and service development projects.
	**41.**	e. Establish and monitor quality indicators to demonstrate the effectiveness of mental health pharmacy services.
	**42.**	f. Teach clinical pharmacy in mental health, including within undergraduate and postgraduate programmes, and among pharmacists and other healthcare professions.
	**43.**	g. Supervise undergraduate research, including thesis/dissertation supervision.
	**44.**	h. Conduct projects in mental health pharmacy to support innovation, service development, and reimbursement models.
	**45.**	i. Disseminate results associated with service evaluation to relevant stakeholders, including healthcare leaders, policymakers, and the public.
