# Supplementary material for: Developing a core competency framework for psychiatric pharmacists: a Europe-wide e-Delphi consensus study conducted by the European Society of Clinical Pharmacy Mental Health Special Interest Group
Source: Int J Clin Pharm. 2026 Aug 28;48(6):2360–72. doi: 10.1007/s11096-026-02213-0 (PMC13731694; doi:10.1007/s11096-026-02213-0)
Supplement: Supplementary file 2 [file 11096_2026_2213_MOESM2_ESM.pdf]

Supplementary Material S2. Full text of the Round 1 Delphi questionnaire

Delphi Study: Key Activities and Roles of Clinical Pharmacists Working in Mental Health

This supplementary material reproduces the question and response-option text used in Round 1 of the Delphi study. No participant-level response data are included.

Response scale reference

No.	Question / field	Response format
1	Agreement scale	Strongly agree / Agree / Disagree / Strongly disagree
2	Feasibility scale	Highly feasible / Feasible / Moderately feasible / Low feasibility / Not feasible at all
3	Frequency scale	Always (daily or almost daily) / Often (weekly) / Sometimes (monthly) / Rarely (less than monthly) / Never
4	Priority scale	High priority / Medium priority / Low priority
5	Multiple choice	Several options could be selected where indicated.
6	Single choice	One option could be selected where indicated.
7	Open text	Free-text response.

Participant information and eligibility

No.	Question / field	Response format
1	Do you voluntarily agree to participate in this Delphi study under the conditions described above?	Yes/No
2	Code (provided in email)	Numeric / open response
3	Age (years)	Numeric / open response
4	Country of practice	Open text / single choice
5	What is your highest completed degree?	Open text / single choice
6	How many years of professional experience do you have in pharmacy?	Single choice
7	How many years of experience do you have in clinical pharmacy in psychiatry?	Single choice
8	If you do not work in mental health pharmacy full-time, what percentage of your current work is dedicated to psychiatry?	Single choice
9	What is your primary workplace/practice setting?	Open text / single choice
10	Are you actively involved in research?	Yes/No
11	How many peer-reviewed publications have you contributed to in the last 5 years (in clinical pharmacy or psychiatry field):	Single choice
12	Participation in committees/working groups relating to mental health pharmacy (tick all that apply):	Multiple choice
13	Membership in professional societies relating to mental health pharmacy (tick all that apply):	Open text / multiple choice
14	Role in education and training (tick all that apply):	Multiple choice
15	Typical involvement:	Single choice

Domain 1: Medication review, reconciliation, optimisation and high-risk psychotropics

No.	Question / field	Response format
1	a. Conduct comprehensive medication reviews for individuals with mental illness including pharmacotherapy across specialties. This includes reviewing the appropriateness of the following prescribing parameters relating to each medication: (i) indication, including optimised selection within the drug class; (ii) duration of treatment; (iii) potential for drug-drug and drug-disease interactions; (iv) proactive management, including prevention of adverse drug reactions and other medicine-related problems.	Agreement scale
2	b. Prioritise structured medication review in older adults and others with multimorbidities (e.g., cardiac, renal, or hepatic impairment), polypharmacy, frailty, or use of higher-risk psychotropic medicines (e.g., high-dose antipsychotics, lithium, anticholinergic drugs, benzodiazepines) to reduce treatment burden, prevent medication-related harm, and improve quality of life.	Agreement scale
3	c. Use validated tools (e.g., Beers, STOPP/START) within comprehensive medication reviews to recommend safer alternatives and minimise the risk of prescribing cascades.	Agreement scale
4	d. Provide medication reviews within both primary and secondary care settings.	Agreement scale
5	e. Ensure medication reconciliation is completed. Medicines reconciliation includes ensuring an accurate and complete medication history is taken, identifying and resolving discrepancies, and reviewing pharmacotherapy in collaboration with the multidisciplinary team. Reconciliation may be repeated in community, primary care settings, and at transition points with follow-up reviews added as needed to ensure safe and coordinated care delivery.	Agreement scale
6	f. Provide seamless care, including during admission and transitions of care, including discharge.	Agreement scale
7	g. Support adherence through tailored interventions and facilitate shared decision-making within consultations (e.g., support with long-acting injectable antipsychotic administration, management of adverse effects, etc.).	Agreement scale
8	h. Manage clinically significant drug interactions (including drug-drug, drug-gene, and drug-disease, etc.)	Agreement scale
9	i. Optimise use of high-risk psychotropics (including clozapine and long-acting injectable antipsychotics/high-dose antipsychotics), including adherence to physical health monitoring requirements.	Agreement scale
10	j. Optimise the prescribing of psychotropic medication using pharmacokinetic and pharmacogenomic tools, as appropriate.	Agreement scale
11	k. Make therapeutic substitutions where appropriate (e.g., formulary limitations, medicine shortages) ensuring equivalent efficacy and safety.	Agreement scale
12	l. Provide interventions at multiple points of care, including admission and transitions of care, including discharge.	Agreement scale
13	1. Overall feasibility in your setting<br>How feasible is it to implement the activities in this domain in your current setting?	Single choice: feasibility scale
14	2. Current implementation frequency<br>How often are activities in this domain currently implemented in your practice?	Single choice: frequency scale
15	3. Priority for the next 12 months<br>How would you prioritise this domain for development in the next 12 months?	Single choice: priority scale
16	4. Main barrier (multiple-choice)<br>What is the main barrier to implementing this domain in your practice?	Multiple choice/open text
17	5. Who mainly performs these activities currently? (multiple-choice)	Multiple choice/open text
18	Comment (optional):<br>Do you feel that any important aspects are missing from this domain, or that any items require clarification or refinement?	Open text

Domain 2: Physical health, lifestyle and somatic comorbidities

No.	Question / field	Response format
1	a. Recognise and address physical–mental health interdependencies (e.g., depression linked to hormonal imbalance) and recommend appropriate interventions (e.g., tests or reviews).	Agreement scale
2	b. Conduct physical health checks for people on psychotropic medication, including weight, waist circumference, blood pressure, pulse, and necessary blood tests at recommended intervals, and act on any out-of-range results (e.g., provide appropriate management, recommend referral to an alternative clinician).	Agreement scale
3	c. Recommend and implement physical health and lifestyle interventions for people with mental illness, including smoking cessation services, weight management, preventive screening programmes, and protocols supporting individuals with somatic comorbidities or medication-related risks (e.g., clozapine).	Agreement scale
4	d. Use relevant tools to support recommendations for physical health interventions (e.g., the Lester Tool) and modifiable lifestyle risk factors (such as smoking and substance use).	Agreement scale
5	e. Monitor chronic diseases in at-risk individuals to minimise the impact of psychotropic medication on disease burden and to support optimisation of chronic conditions (e.g., diabetes, hypertension or obesity).	Agreement scale
6	1. Overall feasibility in your setting<br>How feasible is it to implement the activities in this domain in your current setting?	Single choice: feasibility scale
7	2. Current implementation frequency<br>How often are activities in this domain currently implemented in your practice?	Single choice: frequency scale
8	3. Priority for the next 12 months<br>How would you prioritise this domain for development in the next 12 months?	Single choice: priority scale
9	4. Main barrier (multiple-choice)<br>What is the main barrier to implementing this domain in your practice?	Multiple choice/open text
10	5. Who mainly performs these activities currently? (multiple-choice)	Multiple choice/open text
11	Comment (optional):<br>Do you feel that any important aspects are missing from this domain, or that any items require clarification or refinement?	Open text

Domain 3: Person-centred care, counselling and psychoeducation

No.	Question / field	Response format
1	a. Provide personalised and person-centred pharmaceutical care, including involving people and their families or carers as active partners in planning, monitoring, and decision-making throughout treatment.	Agreement scale
2	b. Offer clear medication counselling and psychoeducation to people and their families or carers, supporting understanding, adherence, and shared treatment goals while accommodating individual cognitive or health literacy needs.	Agreement scale
3	1. Overall feasibility in your setting<br>How feasible is it to implement the activities in this domain in your current setting?	Single choice: feasibility scale
4	2. Current implementation frequency<br>How often are activities in this domain currently implemented in your practice?	Single choice: frequency scale
5	3. Priority for the next 12 months<br>How would you prioritise this domain for development in the next 12 months?	Single choice: priority scale
6	4. Main barrier (multiple-choice)<br>What is the main barrier to implementing this domain in your practice?	Multiple choice/open text
7	5. Who mainly performs these activities currently? (multiple-choice)	Multiple choice/open text
8	Comment (optional):<br>Do you feel that any important aspects are missing from this domain, or that any items require clarification or refinement?	Open text

Domain 4: Multidisciplinary collaboration, communication and professional engagement

No.	Question / field	Response format
1	a. Integrate into multidisciplinary teams to provide expert pharmaceutical input	Agreement scale
2	b. Collaborate across care settings with pharmacists (hospital, community, general practice) and other healthcare professionals (e.g., psychiatrists, GPs, nurses, geriatricians, social workers, occupational therapists) to ensure safe and effective treatment and continuity of care, particularly during transitions of care (including admission, discharge, and primary care).	Agreement scale
3	c. Document and communicate care plans, actions, and outcomes in a timely manner to support collaborative decision-making among the team.	Agreement scale
4	d. Engage in professional networks and representative groups at local, national, and international levels to support service development and professional advocacy.	Agreement scale
5	e. Contribute to discussions on reimbursement models by working with commissioners, insurers and within healthcare systems to demonstrate the value of pharmacy services.	Agreement scale
6	1. Overall feasibility in your setting<br>How feasible is it to implement the activities in this domain in your current setting?	Single choice: feasibility scale
7	2. Current implementation frequency<br>How often are activities in this domain currently implemented in your practice?	Single choice: frequency scale
8	3. Priority for the next 12 months<br>How would you prioritise this domain for development in the next 12 months?	Single choice: priority scale
9	4. Main barrier (multiple-choice)<br>What is the main barrier to implementing this domain in your practice?	Multiple choice/open text
10	5. Who mainly performs these activities currently? (multiple-choice)	Multiple choice/open text
11	Comment (optional):<br>Do you feel that any important aspects are missing from this domain, or that any items require clarification or refinement?	Open text

Domain 5: Advanced clinical services, prescribing and digital tools

No.	Question / field	Response format
1	a. Undertake dependent (collaborative) prescribing under shared care agreements with physicians.	Agreement scale
2	b. Undertake independent prescribing within a defined scope of practice.	Agreement scale
3	c. Develop pharmacist-led services for people with mental illnesses, including support for more severe and enduring mental illnesses (e.g., clozapine clinics).	Agreement scale
4	d. Provide direct behavioural or mental health support in exceptional circumstances (e.g., crisis situations), where additional specialist training is available.	Agreement scale
5	e. Facilitate group-based interventions (e.g., therapeutic groups for people with mental illnesses) to encourage discussion and identify medication-related problems.	Agreement scale
6	f. Offer community post-discharge services, including medicines reconciliation, outpatient medication reviews, and adherence support.	Agreement scale
7	g. Optimise the use of new technologies to support medication-related decision-making (e.g., clinical decision-support systems, electronic prescribing tools, medication safety software, pharmacogenomic decision tools).	Agreement scale
8	1. Overall feasibility in your setting<br>How feasible is it to implement the activities in this domain in your current setting?	Single choice: feasibility scale
9	2. Current implementation frequency<br>How often are activities in this domain currently implemented in your practice?	Single choice: frequency scale
10	3. Priority for the next 12 months<br>How would you prioritise this domain for development in the next 12 months?	Single choice: priority scale

11	4. Main barrier (multiple-choice)<br>What is the main barrier to implementing this domain in your practice?	Multiple choice/open text
12	5. Who mainly performs these activities currently? (multiple-choice)	Multiple choice/open text
13	Comment (optional):<br>Do you feel that any important aspects are missing from this domain, or that any items require clarification or refinement?	Open text

Domain 6: Medication safety, governance, formulary work and implementation of guidance

No.	Question / field	Response format
1	a. Manage referrals and monitor high-risk medications to ensure safe use.	Agreement scale
2	b. Conduct audits of prescribing practices and adherence to best practice standards.	Agreement scale
3	c. Contribute to formulary decision-making regarding approved medications.	Agreement scale
4	d. Develop and review clinical practice guidelines, clinical pharmacy guidance, and decision-making tools to support safe and effective prescribing in mental health.	Agreement scale
5	e. Implement local and national practice recommendations, where relevant (e.g., medication-related monitoring protocols) across clinical teams and within the service.	Agreement scale
6	1. Overall feasibility in your setting<br>How feasible is it to implement the activities in this domain in your current setting?	Single choice: feasibility scale
7	2. Current implementation frequency<br>How often are activities in this domain currently implemented in your practice?	Single choice: frequency scale
8	3. Priority for the next 12 months<br>How would you prioritise this domain for development in the next 12 months?	Single choice: priority scale
9	4. Main barrier (multiple-choice)<br>What is the main barrier to implementing this domain in your practice?	Multiple choice/open text
10	5. Who mainly performs these activities currently? (multiple-choice)	Multiple choice/open text
11	Comment (optional):<br>Do you feel that any important aspects are missing from this domain, or that any items require clarification or refinement?	Open text

Domain 7: Research, education, quality improvement and service development

No.	Question / field	Response format
1	a. Lead or participate in research, including clinical trials relevant to pharmacy services in mental health.	Agreement scale
2	b. Collaborate within academic and professional networks to conduct research relevant to mental health pharmacy.	Agreement scale
3	c. Provide education and mentorship to advance evidence-based mental health pharmacy practice.	Agreement scale
4	d. Undertake quality improvement and service development projects.	Agreement scale
5	e. Establish and monitor quality indicators to demonstrate the effectiveness of mental health pharmacy services.	Agreement scale
6	f. Teach clinical pharmacy in mental health, including within undergraduate and postgraduate programmes, and among pharmacists and other healthcare professions.	Agreement scale
7	g. Supervise undergraduate research, including thesis/dissertation supervision.	Agreement scale
8	h. Conduct projects in mental health pharmacy to support innovation, service development, and reimbursement models.	Agreement scale
9	i. Disseminate results associated with service evaluation to relevant stakeholders, including healthcare leaders, policymakers, and the public.	Agreement scale

10	1. Overall feasibility in your setting<br>How feasible is it to implement the activities in this domain in your current setting?	Single choice: feasibility scale
11	2. Current implementation frequency<br>How often are activities in this domain currently implemented in your practice?	Single choice: frequency scale
12	3. Priority for the next 12 months<br>How would you prioritise this domain for development in the next 12 months?	Single choice: priority scale
13	4. Main barrier (multiple-choice)<br>What is the main barrier to implementing this domain in your practice?	Multiple choice/open text
14	5. Who mainly performs these activities currently? (multiple-choice)	Multiple choice/open text
15	Comment (optional):<br>Do you feel that any important aspects are missing from this domain, or that any items require clarification or refinement?	Open text
