# Supplementary material for: Developing a core competency framework for psychiatric pharmacists: a Europe-wide e-Delphi consensus study conducted by the European Society of Clinical Pharmacy Mental Health Special Interest Group
Source: Int J Clin Pharm. 2026 Aug 28;48(6):2360–72. doi: 10.1007/s11096-026-02213-0 (PMC13731694; doi:10.1007/s11096-026-02213-0)
Supplement: Supplementary file 3 [file 11096_2026_2213_MOESM3_ESM.pdf]

Supplementary Material S3. Item-Level Revisions and Delphi Results Between Rounds 1 and 2

Item ID	Domain	Original Item (R1)	Revised Item (R2)	Reason	Agreement (%) – R1	Median – R1	IQR – R1	Agreement (%) – R2	Median – R2	IQR – R2
2a	Physical Health Monitoring and Interventions	Recognise and address physical–mental health interdependencies (e.g., depression linked to hormonal imbalance) and recommend appropriate interventions (e.g., tests or reviews).	Reworded item 2a. Recognise and flag physical–mental health interdependencies relevant to pharmacotherapy (e.g., medication-related metabolic effects) and recommend appropriate assessment, review, or referral within the multidisciplinary team.	Clarified scope and relevance to pharmacotherapy; emphasised multidisciplinary collaboration.	65.63	3	2	96.30	4	1
2d	Physical Health Monitoring and Interventions	2d. Use relevant tools to support recommendations for physical health interventions (e.g., the Lester Tool) and modifiable lifestyle risk factors (such as smoking and substance use).	Reworded item 2d. Use relevant tools, where available, to support identification of physical health risks and to guide recommendations for modifiable lifestyle factors.	Improved clarity and generalisability; reduced prescriptiveness (e.g., removal of specific tools); enhanced applicability across settings.	62.50	3	2	92.59	3	1
2e	Physical Health Monitoring and Interventions	2e. Monitor chronic diseases in at-risk individuals to minimise the impact of psychotropic medication on disease burden and to support optimisation of chronic conditions (e.g., diabetes, hypertension or obesity).	Reworded item 2e. Support monitoring and optimisation of chronic conditions in people receiving psychotropic medication by identifying medication-related risks, prompting appropriate follow-up, and collaborating with the multidisciplinary team.	Clarified role as supportive and collaborative; emphasised medication-related risk identification and multidisciplinary follow-up.	75.00	3	1	96.30	4	1
3a	Person-Centred Care and Counselling	3a. Provide personalised and person-centred pharmaceutical care, including involving people and their families or carers as active partners in planning, monitoring, and decision-making throughout treatment.	Reworded item 3a. Provide person-centred pharmaceutical care in relation to medicines, involving individuals (and, where appropriate, families or carers) in shared decision-making about pharmacotherapy within the multidisciplinary team.	Clarified the pharmacist's role as supportive and collaborative; emphasised medicines-focused care and shared decision-making within the multidisciplinary team.	71.88	3	2	92.59	3	1
3b	Person-Centred Care and Counselling	3b. Offer clear medication counselling and psychoeducation to people and their families or carers, supporting understanding, adherence, and shared treatment goals while accommodating individual cognitive or health literacy needs.	Reworded item 3b. Provide medication counselling and medicines-related education to the people using them, including families or carers, supporting understanding, adherence, and shared treatment goals, while respecting individual cognitive and health literacy needs.	Improved clarity and focus on medicines-related education; emphasised individualisation according to cognitive and health literacy needs.	71.88	3	2	96.30	3	1
4e	Interprofessional Collaboration and Care Coordination	4e. Contribute to discussions on reimbursement models by working with commissioners, insurers and within healthcare systems to demonstrate the value of pharmacy services.	Reworded item 4e. Contribute, where relevant and feasible, to discussions on funding or reimbursement models to demonstrate the value of mental health pharmacy services within the healthcare system.	Clarified scope and feasibility; broadened the wording to include both funding and reimbursement models and improved applicability across healthcare systems.	68.75	3	2	81.48	3	1
5a	Advanced and Extended Roles	5a. Undertake dependent (collaborative) prescribing under shared care agreements with physicians.	Reworded item 5a. Undertake dependent (collaborative) prescribing under clearly defined shared-care agreements with physicians, where permitted by national legislation and supported by appropriate governance, training, and clinical protocols.	Clarified that dependent prescribing is conditional on national legislation and requires appropriate governance, training, and clinical protocols.	65.63	3	1	59.26	3	1
5c	Advanced and Extended Roles	5c. Develop pharmacist-led services for people with mental illnesses, including support for more severe and enduring mental illnesses (e.g., clozapine clinics).	Reworded item 5c. Develop and contribute to pharmacist-led services for people with mental health conditions, where feasible and appropriately resourced (e.g., clozapine clinics), in collaboration with the multidisciplinary team.	Reduced prescriptiveness by acknowledging variation in feasibility and resources and emphasised collaboration with the multidisciplinary team.	68.75	3	2	70.37	3	2
5e	Advanced and Extended Roles	5e. Facilitate group-based interventions (e.g., therapeutic groups for people with mental illnesses) to encourage discussion and identify medication-related problems.	Reworded item 5e. Facilitate medicines-focused group-based interventions (e.g., education about psychotropic medicines, adherence support, and management of adverse effects), where appropriate within scope of practice.	Clarified the medicines-related focus of the intervention and limited the activity to the pharmacist's scope of practice.	68.75	3	1	92.59	4	1
